# An Evaluation of the Role of an Intermediate Care Facility in the Continuum of Care in Western Cape, South Africa

**DOI:** 10.15171/ijhpm.2017.52

**Published:** 2017-05-14

**Authors:** Sikhumbuzo A. Mabunda, Leslie London, David Pienaar

**Affiliations:** ^1^Public Health Department, Walter Sisulu University, Mthatha, South Africa.; ^2^School of Public Health and Family Medicine, University of Cape Town, Cape Town, South Africa.; ^3^Western Cape Department of Health, Cape Town, South Africa.

**Keywords:** Subacute Care, Intermediate Care (IC), Step-Down Facilities, Stroke Rehabilitation, Continuity of Care, Care-Plan

## Abstract

**Background:** A comprehensive primary healthcare (PHC) approach requires clear referral and continuity of care pathways. South Africa is a lower-middle income country (LMIC) that lacks data on the role of intermediate care (IC) services in the health system. This study described the model of service provision at one facility in Cape Town, including reason for admission, the mix of services and skills provided and needed, patient satisfaction, patient outcome and articulation with other services across the spectrum of care. ****

**Methods:** A multi-method design was used. Sixty-eight patients were recruited over one month in mid-2011 in a prospective cohort. Patient data were collected from clinical record review and an interviewer-administered questionnaire, administered shortly after admission to assess primary and secondary diagnosis, referring institution, knowledge of and previous use of home based care (HBC) services, reason for admission and demographics. A telephonic questionnaire at 9-weeks post-discharge recorded their vital status, use of HBC post-discharge and their satisfaction with care received. Staff members completed a self-administered questionnaire to describe demographics and skills. Cox regression was used to identify predictors of survival.

**Results:** Of the 68 participants, 38% and 24% were referred from a secondary and tertiary hospital, respectively. Stroke (35%) was the most common single reason for admission. The three most common reasons reported why care was better at the IC facility were staff attitude, the presence of physiotherapy and the wound care. Even though most patients reported admission to another health facility in the preceding year, only 13 patients (21%) had ever accessed HBC and only 25% (n=15) of discharged patients used HBC post-discharge. Of the 57 patients traced on follow-up, 21(37%) had died. The presence of a Care-plan was significantly associated with a 62% lower risk of death (hazard ratio: 0.38; CI 0.15–0.97). Notably, 46% of staff members reported performing roles that were outside their scope of practice and there was a mismatch between what staff reported doing and their actual tasks.

**Conclusion:** Clients understood this service as a caring environment primarily responsible for rehabilitation services. A Care-plan beyond admission could significantly reduce mortality. There was poor referral to and poor articulation with HBC services. IC services should be recognised as an integral part of the health system and should be accessible.

## Background

More than 3 decades have passed since the Alma–Ata conference outlinedthe primary healthcare (PHC) approach as a strategy for equitable and appropriate healthcare.^1,2^ Key elements of the PHC approach are: (*a*) it is not limited to curative services but is also inclusive of preventive and rehabilitative care; (*b*) services should prioritise those most in need; and (*c*) to provide comprehensive healthcare, it “…should be sustained by integrated, functional and mutually supportive referral systems….”^1^ Unlike the concept of primary care, with which it is often confused,^1-4^ the broader conception of PHC describes an approach to the organisation of the health system that includes services delivered to individuals (primary care services) and public health-type functions.^3^

A PHC approach will therefore not only enhance an equitable access to health services but will also ensure continuity of care in the health system at all stages in the care pathway.^1,3^ This is consistent with the White Paper for the Transformation of the Health System of South Africa’s first democratic government, which aimed to develop a unified health system^5^ and with South Africa’s 2008 “Discussion Document On The Development Of Subacute Policy Guideline”^6^ which confirmed the idea that a continuum of care includes both acute and subacute modalities.^6^ It is generally agreed, therefore, that subacute care (SAC) should be seen as an extension of hospital care concerned with rehabilitation.^7^

A challenge in evaluating SAC is that institutions providing similar service may be given different names across the world and those models and packages of care vary depending on the location.^8-14^ For example, in the United States, SAC may be interchangeably described as Postacute Care,^8^ accessed in a wide range of settings that include skilled nursing facilities (SNFs), inpatient rehabilitation facilities (IRFs), long-term care hospitals (LTCHs) or the care delivered by home health agencies (HHAs) in patients’ residences.^8^ These services differ in the extent of multidisciplinary therapy, the duration and nature of nursing care and the form of a medical services.^9^

In the United Kingdom, SAC facilities are known as “intermediate care facilities”^13,14^ and are designed for patients who would otherwise face a prolonged hospital stay at acute hospitals. Admission is limited to 6 weeks, has a planned outcome of maximising independence and is rendered by a multi-disciplinary care team.^13^ Such facilities include geriatric day hospitals; hospital-at-home schemes and home-based rehabilitation; community hospitals; rapid response teams; community assessment and rehabilitation teams; nurse-led units; social service schemes; day centre rehabilitation; and residential care rehabilitation.^13^

The Australian SAC model “…includes rehabilitation, palliative care, psychogeriatric care, geriatric evaluation and management; and maintenance care.”^15^ This type of care, offered either on an inpatient or outpatient basis, can take place as a substitute for acute hospitalisation or directly after discharge from acute hospitalisation.^15^

All these models have in common the concept that care focuses on enhancing the “…quality of life and/or functional status”^15^ and is generally delivered by multi-disciplinary care teams, at intensity lower than that provided in acute facilities.^13-16^ The South African health system further incorporates home-based care (HBC) services in the form of community health workers in the district health system (DHS).^6^ This level of care ensures that patients who need access to healthcare can receive health services from home without being admitted into hospital or SAC.^6,7^ It is therefore logical that patients with a chronic illness and/or those with a discharge from a SAC facility will be the main beneficiaries of HBC.

There is, however, a paucity of research on SAC in Lower-Middle Income Countries (LMICs), where the challenges for health systems are substantially different. For example, in the South African context, factors which have shaped SAC include the growth of the HIV epidemic, changing approaches to the management of HIV/AIDS (including universal access to treatment programmes) and the more recent recognition of the increasing burden of non-communicable diseases (NCDs).^17-20^ This has necessitated a shift towards ensuring service delivery platforms able to manage many chronic lifelong conditions.^17,18,20-22^

In 2012, the Western Cape (WC) provincial Department of Health (DoH) undertook a review of its SAC policy,^21,22^ and a task team report led to the development of an intermediate care (IC) policy framework in September 2012, which revised the organisation of what was delivered as a SAC programme in the health department.^21,22^ IC broadly included patients requiring post-acute care, restorative and rehabilitative, and palliative care. The policy, however, proposed not only a broader and more comprehensive definition of the services offered in de-hospitalised care but also the introduction of a new cadre of mid-level workers known as rehabilitation care workers (RCWs).^21,22^

There are few studies published in South Africa or the African region on SAC or any IC component, such as step-down facilities and chronic lifelong care centres. Where SAC-related research is available in South Africa, it usually focuses on HIV-related HBC.^23-27^ There is little evidence on which to base policies on community-based services (CBS) and SAC models in South Africa.^6,7,21,22^

This study was therefore initiated in response to the strategic opportunity identified by the WC DoH for IC to improve the efficiency of the health system, and to provide data for planning an efficient DHS based on a PHC approach. While SAC is the term commonly used in the international literature, this study uses the definition of IC used in the policy.^21,22^ In this policy IC is described as an intergrated provision of inpatient sub-acute, stepdown, respite, palliative and some chronic services.^21,22^

The study describes the model of service provision of a single large IC facility in Cape Town and its role in the continuum of care. This was achieved through characterising (*i*) patient demographics and reason for admission; (*ii*) patient care needs in relation to the skills of staff who provide IC; (*iii*) duration of stay in the facility; (*iv*) patient and staff understanding of IC; (*v*) patient outcomes; (*vi*) survival at follow up and the; (*vii*) availability of family support. We also explored whether age, a stroke diagnosis at admission, the presence of a Care-plan at discharge and a confirmed community health worker visit were associated with survival amongst IC patients.

This study defines continuum of care as the patient journey within the health system and how services relate to each other; and acknowledging that the scope of this study can only track the journey from a referring hospital to a community-based service.

## Methods

### Study Setting

The DHS in the WC comprises 6 Districts, five rural and one located in urban Cape Town. More than 65% of the WC population reside within the Cape Town Metro District,^28^ which is further divided into 8 sub-districts paired to form 4 substructures. It is to the substructure level that governance powers are decentralised.

At the time of the study the WC had 9 SAC facilities, which were located in four of the 6 districts (3 rural and the Cape Town Metro). After the IC policy review and the change in definition of SAC the WC province was noted to have 25 IC facilities.^21,22^ The biggest IC facility in Cape Town designated for subacute and palliative care services delivered by a wide range of health professionals, was selected for this study. This IC facility is a private not-for-profit hospital contracted to deliver comprehensive services to the public with 106 beds: 84 beds allocated to subacute services/Subacute Care Ward (SACW) and 22 beds allocated to respite/palliative care, respectively. The SACW housed patients needing respite, convalescent and rehabilitative care.

The Health Department described IC as comprising “…integrated provision of inpatient services formerly referred to as sub-acute, step down, respite, palliative, and some of chronic care under de-hospitalised care services.”^21^ Of the three basic models described in the WC Intemediate Care policy document (IC service within an acute hospital, IC service on acute hospital premises and IC service not on acute hospital services), the study facility followed the latter model where non-profit organisation staff provide services with limited support by the DoH involving payment of a medical doctor’s salary.^21,22^

### Study Design, Population and Sampling

The study used a multi-method approach to capture provider and patient factors in IC. Firstly, to assess patient factors, a prospective cohort study was conducted with clients admitted to IC between the 27th of June and 26th of July 2011.

Based on the fact that the facility had a bed utilisation rate of ±100% for the 106 beds, the study aimed to recruit at least 100 patients into the study using purposive sampling. Data were collected during the course of their admission in IC using (*i*) a record review and (*ii*) an interviewer-administered questionnaire administered shortly after admission and at follow-up in the respondent’s preferred language (English, isiXhosa or Afrikaans).

During admission, arrangements were made for post-discharge follow-up with clients, family member(s) and/or caregivers by securing a telephonic contact number. The follow-up comprised an interviewer-administered questionnaire administered by telephone at an average of 9 weeks (range 5-13 weeks) post-discharge.

Secondly, a cross-sectional survey of all staff members employed at first of May 2011 was conducted, using a self-administered questionnaire. Inclusion criteria were that staff (*i*) worked directly with patients at the facility, and (*ii*) had been employed at the facility for a minimum of two months prior to the study (June 27, 2011), and (*iii*) delivered clinical services.

### Measurements

#### Record Review Instrument

Data on diagnoses, medication and the patient’s Care-plan were abstracted from medical records using a standardised data capture form at the same time as the interviews. The form also recorded any specific treatment plans noted by any of the different providers. In instances where clinical information and/or treatment plans were not clear on admission, the referring institutions were consulted for clarification.

The record review provided an inventory of the range of care services provided at the institution. There was no limit on the number of care services recorded for each patient. These were inductively coded into themes in a process of post-coding.

#### Client Questionnaire at Intermediate Care Facility

The client questionnaire collected data during IC facility admission and included elements adapted from the International Classification of Functioning, Disability and Health (ICF).^29^ The ICF^29^ is a standardised and validated instrument that measures health, disease and disability across cultures. The clinical folder was considered to be the more reliable source of clinical information if there was any discrepancy between patient and folder information.

#### Record Review Post-discharge

The discharge plan information captured from the clinical folder at discharge included the place to which a patient would be discharged, contact information, evidence of referral to HBC and presence of a Care-plan at admission and at discharge. A Care-plan is defined as a plan detailing future care a patient should receive. We identified 2 Care-plans; one issued by the referring institution for the duration of admission in the IC (called Referring institution Care-plan); and another issued by the IC for care after discharge in the home and community environment (called IC Discharge Care-plan).

#### Client Questionnaire Post-discharge

A telephonic follow-up interview was conducted to assess the client’s vital status (alive or dead), clinical condition (improved or deteriorated), satisfaction with care received at the IC facility and referring institution and whether they accessed any other health services since discharge from IC.

#### Staff Questionnaire

A standardised instrument was designed and piloted, exploring demographic factors, skills and competencies so as to enable characterisation of patient care needs in relation to the skills of staff who provide IC. Respondents were asked about their skills and about patient care needs that take up most of their time and could name as many skills or needs as they felt important (Table 1). Staff skills and patient care needs were categorised according to the same six themes identified in the record review, as described above. Themes included (*i*) activities of daily living (ADL); (*ii*) Nursing care; (*iii*) Specialised care (IC specific); (*iv*) Rehabilitation; (*v*) Administration; and (*vi*) Social work and other. These 6 themes, combined with data from the staff survey (see below) formed a template for assessing care provided to patients (Table 1).

**Table 1:** Staff Skills and Patient Needs as Reported by Staff and as Recorded on Clinical Notes

**ADL**	**Nursing Care**	**Rehabilitation**	**Specialised Care**	**Administration**	**Social Work**
Bath patient	Oxygen administration	Manual patient handling	Catheter insertion	Administration	Translator
Toileting	Suture removal	Mobilise patient	Administer medication	Admissions	Communication skills
Shaving	Subcutaneous insulin	Wheel-chair training	Dispensing	Discharging	Walk patient
ADL assessment	Catheter care including emptying urine bag	Active exercise	Assessment	Teaching	Socialise with patient
Nail care	Catheter removal	Exercise	Intravenous line insertion	Supervision	Family participation
Hair care	Vital signs (BP, temperature, input and output chart)	Passive exercise	Wound care (including wound dressing and sterile techniques)	Checking medication	Liaise with family
Turning patient	Urinalysis	Groupwork	Trauma	Medication ward stock	Disability grant application
Bed making	Tube feeding	Voice exercise	Triage	Delegation of duties	Identity document application
Feeding	Insulin (subcutaneous)	Movement training	Midwifery	Meeting procedures	Finding family
Dressing and undressing patient	Suctioning (including tracheostomy care)	Bed-to-chair transfer	Control drugs	Training of subordinates	Geriatric care
ADL	Nursing care	Posture correction	Intravenous line maintenance	Stock control	Placement in social services or institutions of care (eg, old age home)
Pressure care	Health education	Bed mobility	HIV management	Stock replenishment	Family meeting
Hygiene	Diet	Speech	Minor Surgical procedures (eg, abscess management)	Order medication	Sexual abuse counselling
Socialise with patient	Counselling (includes knowledge of patient’s condition)	Physiotherapy	Diagnosing	Secretarial duties	Drug abuse counselling
Open windows	Patient care	Occupational therapy	Advice on medicines	Referral to other health institutions	Attend to patient’s spiritual needs
Nappy change	Incident report	Speech therapy	Examining patients	Supply drugs to ward	Empathy
Mouth care	Drip aid (ensuring that Intravenous line is running)	Neuro-developmental skills	Medical examinations	Sick notes	Interpersonal skills
	Clean deceased	Trunk exercises	Reporting incidents	Drugs control	Restrain patient
	Hgt monitoring	Use of assistive devices	Infection control	Order stock	Communication
	Hb monitoring	Physical assistance	Gynaecology	Stock assistive devices	Compassionate skills
	Follow orders	Joint mobilisation	Ward round		Group work
	Assist giving medication	Muscle power	Phlebotomy		Bereavement counselling
	First aid	Language stimulation	Daily report		Palliative care
	Tracheostomy care	Rehab education	Child health		
	TLC	Speech augmentation	Intramuscular injection		
	Specimen collection	Dysarthria	Assist doctor during ward round		
	Prepare before procedure	Assist physiotherapy(ist)	Nasogastric tube insertion		
	Wax checking	Movement return	Professional nurses’ duties		
	Escort patient	Massaging	Antiretroviral clinic		
	Plaster of Paris care	Balance	Antiretroviral drug issuing		
		Encourage movement	Medication		
		Creative skills	Discharge plan		
		Sign language	Treatment		
		Communication charts	Assist Assessment		
		Swallow screen	Drug compatibility		
		Slings and splints			
		Subjective assessment			
		Objective assessment			

Abbreviations: ADL, activities of daily living; BP, blood pressure.

### Statistical Analysis

All data collected were analysed in STATA 12.1 (Stata Corp LP, College Station, TX, USA) and Microsoft Excel 2010 (Microsoft Corporation, Seattle, WA, USA). Each patient and staff respondent could generate between 1 and 8 perceived care needs, care type provided or staff skill. Frequencies were estimated for categorical data. Numerical data, such as age (years) that were normally distributed were reported in terms of the mean and range; data that were not normally distributed such as LoS (days) were reported in terms of median and interquartile range (IQR). A 95% CI was used to report on the precision of estimates. Three hypotheses were tested: (*a*) A Care-plan beyond admission in IC improves survival; (*b*) There is a poor referral system between HBC and IC services; (*c*) Patients in IC mostly require assistance with ADL. The level of significance for hypothesis testing was set at 5% (*P* < . 05).

For normally distributed data with equal variances, the 2-sample *t* test was used to compare continuous variables; otherwise the Wilcoxon sum-rank test was used. The Wilcoxon signed rank test was used to compare the LoS between the referring institution and IC as this variable was not normally distributed.

The Kaplan–Meier survival method was used to estimate the time of survival in the study. Cox’s proportional hazard model was used to predict the relationship between survival and independent predictors (Care-plan, age and stroke diagnosis).

The Aikaike information criterion was used to test the validity of the models and the Schoenfield residuals were used to test whether overall proportional hazard assumption was met.

## Results

### Patient Profile and Admission History

All patients admitted through the palliative care section did not pay for their hospitalisation because of a complete subsidy. Those admitted to the SACW were expected to pay about US$2.5 per day but this was waived if they were unemployed or indigent. The hospital also provides transport at a fee for patients requiring follow up appointments at the referring hospital during their stay. This fee ranged from about US$11 for the closest facilities to US$22 for transportation to the furthest facility.

**Figure 1:**
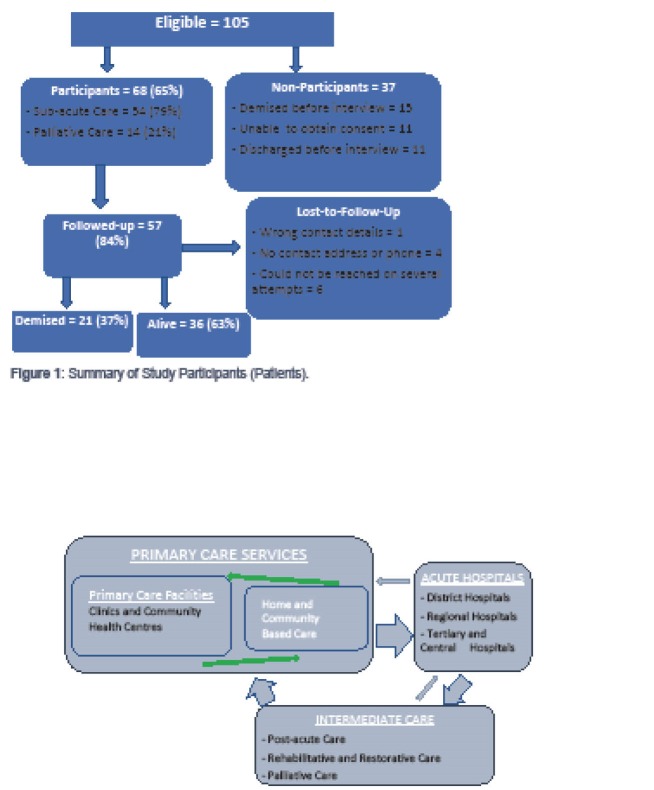
Summary of Study Participants (Patients).

Of the 105 patients who were admitted in the IC facility between the 27th of June to the 26th of July 2011, 68 (65%; CI: 55%–74%) were interviewed (Figure 1). Non-participants included 11 patients (10%) who met the study’s inclusion criteria, but were not medically fit to give consent and whose families could not be contacted; 15 patients who were admitted in the study time period but died before first interview (14%); and 11 patients (10%) discharged before being interviewed. Amongst those who were eligible, there were no refusals in both the initial and the follow-up interview.

The median interval between admission at IC and first interview was 11 days. The LoS at their first institution prior to referral to IC was significantly shorter (*P* < .01; median 14 days; IQR of 8-23 days and a maximum of 62 days) than their stay in IC where the median LoS was 31 days (IQR 17.5–41 days; with a maximum stay of 111 days or ±16 weeks).

The average duration to participant follow-up post discharge was 60 days (range 35 to 91 days). Eleven participants could not be traced at follow-up resulting in loss-to-follow-up (LTFU) of 16.2%, with the majority of these patients being those who had been admitted in the palliative care section of the SAC facility (n = 7).

Patients were evenly divided between males and females (Table 2); 79% were admitted to the SACW (n = 54) and the other 21% (n = 14) to the palliative care section. Of the 14 patients admitted in the palliative care section, 79% (n = 11) had both HIV/AIDS and tuberculosis.

**Table 2:** Demographic Characteristics of Patients Interviewed

	**IQR** ^*^	**Median (95% CI)**	***P***
Age (y) by gender			
Male	40.90–65.30	54.70 (47–62)	.17^a^
Female	54.30–68.70	62.0 (58–66)	
Total	41.90–67.30	59.0 (55–62)	
Age (y) by admission ward			
SACW	21.90–87.50	60.0 (55.90–64.0)	.01^b^
Palliative ward	22.0–65.20	41.30 (34.10–48.60)	
No. of adults in household	1-3	2	
No. of adults employed in household	0-1	0.50	
Gender	**n (%)**		
Male	36 (53)		.49^c^
Female	32 (47)
Total	68
Marital status			
Single	26 (38)	
Married	19 (28)	
Widowed	14 (22)	
Separated or divorced	7 (10)	
Cohabiting	1 (1)	
Place of residence			
Own home	34 (50)	
Not own home	25 (36.76)	
Institution	5 (7.35)	
Homeless	4 (5.88)	
Presence of carer			
Yes	51 (75)	
No	17 (25)	
Nature of relations with Carer			
First degree relative (spouse, child, parent or sibling)	31 (60.78)	
Other relatives	18 (35.29)	
Friend	2 (3.92)	
Employment			
Paid employment	7 (10.29)	
Retired	28 (41.18)	
Unemployed	30 (44.12)	
Other (self-employed and a student)	3 (4.41)	
Type of grant			
Old age grant (state)	28 (70)	
Disability grant (state)	7 (17.50)	
Employer benefits	3 (7.50)	
Child support grant (state)	2 (2)	

Abbreviations: IQR, interquartile range; SACW, subacute care ward. ^a^ Wilcoxon sum rank test; ^b^ Two sample *t* test; ^c^‏ Two sample test of proportions.

Only 10% (n = 7) of clients were in paid employment. Most were either retired (41%; n = 28), unemployed for health reasons (24%; n = 16) or unemployed because they were unable to find employment (21%; n = 14). Most respondents (n = 40 or 59%) reported receipt of a social grant, such as an old age grant (42%; n = 28), disability grant (10%; n = 7), child support grant (3%; n = 2) and three received employer benefits due to health reasons or retrenchment.

Family support, framed as having a person to care for them, was reported by 75% (n = 51) of clients. An equal number of participants (n = 18 each) reported being cared for by a first degree relative (spouse, child, parent or sibling) and by other relatives. For just over one third (n = 18) of the participants with a carer, the carer was reported as living at a different household to the respondent. The median number of adults living in the respondent’s permanent household was reported as 2 adults (IQR 1–3) and 50% of participants (n = 34) reported to not having an adult employed in the household.

Most participants were residents in the WC (91%; n = 62), primarily from within the Metro District (90%; n = 61); 9% were from the neighbouring Eastern Cape province who had migrated for medical care (Table 3). All patients had been referred from services within the Metro District. All patients were referred from acute hospitals. Most patients had been referred from a secondary hospital (38%; n = 26), tertiary hospital (24%; n = 16) and from 3 district (level 1) hospitals (16%; n = 11) in the same substructure as the IC facility. Only 22% (n = 15) of participants were referred from a substructure other than that in which the IC facility is located. Of all the participants interviewed, 41% (n = 28) reported being hospitalised in the previous year.

**Table 3:** Locality Where Patients Resided Before Being Admitted to SAC by and District, Substructure and Sub-district

**District**	**n (%)**	**Substructure**	**n (%)**	**Sub-district**	**n (%)**
Cape Town (Metro)	61 (90)	Southern-Western	35 (51)	Western	23 (34)
			Southern	12 (18)
		Klipfontein-Mitchells Plain	16 (24)	Klipfontein	9 (13)
				Mitchells Plain	7 (10)
		Tygerberg-Northern	5 (7)	Tygerberg	5 (7)
		Khayelitsha-Eastern	5 (7)	Khayelitsha	3 (4)
				Eastern	2 (3)
Cape Winelands		1 (1)	Witzenberg	1 (1)
Other (Eastern Cape)^a^			6 (9)

Abbreviation: SAC, subacute care ward. ^a^ Other refers to participants from the Eastern Cape province.

Only 53% of respondents (n = 35) admitted to knowing of HBC or community health workers (CHWs); 21% (n = 13) of clients had used HBC at some time in the past; and 5 of the 29 (17%) participants who had been admitted in the previous year had made use of HBC. A Care-plan which extended beyond their admission at IC was present in 69% (n = 47) of patient records.

### Patient Care Needs

Stroke was found to be the most prevalent condition (Table 4) leading to admission (35%; n = 24). Conditions needing an amputation of one or both lower limbs such as cellulitis, peripheral vascular disease (PVD) and other forms of gangrenous limbs were prevalent in 18% (n = 12) of clients interviewed. Tuberculous meningitis and seizures of unknown aetiology were present in 16% (n = 11) of participants.

**Table 4:** Distribution of Conditions Which Led to This Admission at Referral Institution

**Prevalent Primary Conditions**	**No.**	**Percent**
Stroke	24	35
PVD, diabetic ulcer or gangrenous limb	12	18
TB, TB meningitis and seizures of unknown aetiology	11	16
Fracture or osteoarthritis	6	9
Lower respiratory tract Infections	4	6
CCF-pericarditis	3	4
Lung cancer	2	3
Other^a^	6	9
Total	68	100

Abbreviations: TB, tuberculosis; PVD, peripheral vascular disease; CCF, congestive cardiac failure. ^a^ Other includes 1 each of fibrosarcoma, prostate cancer, eclampsia, chronic Kidney disease, bowel perforation and gastroenteritis, PVD, and CCF.

Of the sample, 84% (n = 56) were found to have been diagnosed and on treatment for at least one chronic condition. Hypertension was prevalent in 53% (n = 36) of clients sampled and this was followed by HIV (21%; n = 14), tuberculosis (TB) (19%; n = 13) and diabetes mellitus (DM at 18%; n = 12).

Most clients (85%; n = 58) came to IC using at least one assistive device, usually a wheelchair (n = 39 or 67%), quadripod (n =10 or 17%) or crutches (n = 9 or 16%). Wheelchair use was both temporary during convalescence while others were bedbound. Pressure ulcers were noted in 12% (n = 8) of participants and 75% (n = 6) of these were noted as present before admission to IC. On average, participants were completely unable to carry out usual day-to-day household activities for 16 days in the prior 30-days because of health reasons and they had to reduce or cut back on activities for an average of ±9 days.

When juxtaposing patient care as recorded on patient records with care and skills reported by staff, there were some similarities but also important differences (Table 5). According to the patient record; nursing (82%), specialised care (66%) and rehabilitation care (63%) accounted for the largest number of services received by patients while at IC. When staff were asked about the skills that they believed they have and the patient needs that take up most of their working time, ADLs were reported more frequently than any of the other care categories. Administrative skills were reported as the second least frequent skill possessed by staff and also reported as the lowest patient care need according to staff (Table 5).

**Table 5:** Domains of Patient Needs and Staff Skills Reported by Staff and Identified in Record Review

**Patient Care Needs/Staff Skills**	**Report by staff (n = 70)**		**Patient Record Review (n = 68)**
	**Staff Skills, No. (%)**	**Perceived Patient Care Needs, No. (%)**	**Patient Care Provided, No. (%)**
ADL	49 (70)	57 (81)	35 (51)
Nursing care	39 (56)	36 (51)	56 (82)
Specialised care	44 (63)	40 (57)	45 (66)
Rehabilitation care	20 (29)	26 (37)	43 (63)
Social work and other	10 (14)	16 (23)	9 (13)
Administration	19 (27)	9 (13)	N/A^a^

Abbreviation: ADL, activities of daily leaving. ^a^ Elements are not mutually exclusive, eg, only one ADL was considered if a staff member reported more than one ADL. The same applies for all the other elements for both staff and patients.

Table 5 further shows that staff reported more patient care needs relative to their skills – particularly related to ADLs, rehabilitation, and social work care. When compared with the record review, staff showed a shortfall in rehabilitation skills to meet patient care needs.

### Human Resources

Staffing included a range of healthcare providers. The South African health system recognises 3 nursing categories: (*i*) an enrolled nursing assistant (ENA) with a 6-12 month nursing certificate, (*ii*) an enrolled nurse (EN) with a 2-year nursing Diploma; and (*iii*) a professional nurse (PN) with a 4-year degree or diploma. Caregiver is a category of lay health worker who renders basic care needs in IC. Even though caregivers receive basic training, they do not have professional qualifications. A sessional medical doctor provided on-site care for four hours on every week day and was available for telephonic advice for 24-hours a day on every day of the week. Other staff included a full-time social worker, physiotherapists (PTs) and occupational therapists (OT) working 40-hours per-week and complemented by a speech therapist who was available for 20-hours a week.

There were 85 employees who met the study’s inclusion criteria. Seventy staff members participated, representing a response rate of 82% (CI: 73%–90%). Two staff members refused to participate and other non-respondents (n = 13) were those on leave. Most staff members were female (83%; CI: 9%–28%). The median age was 42 years (IQR 31–54). Male staff were younger (median age 32 years) than female staff (median 43 years; *P* = .02).

Staff comprised of a mix of categories including caregivers (44%; n = 31), nurses (44%; n = 31), medical doctor (n = 1), rehabilitation professionals (7%; n = 5), social work (n = 1) and pharmacy (n = 1) staff. Rehabilitation staff included 2 PTs, a speech therapist, an OT and an OT assistant.

The majority (89.6%; 95% CI: 79.7%–95.7%) felt that IC provided adequate supervision and support for junior staff. With regard to the proposed category of mid-level rehabilitation worker, tasks viewed as appropriate for mid-level workers at IC under supervision included wound care, nasogastric tube (NGT) insertion, catheter insertion and the administration of medication, as well as unsupervised tasks involving ADLs, collection of vital signs eg, temperature reading, administration and the transfer of patients from bed to chair.

Most staff (n = 64 [94.1%; CI: 88.4%-99.9%]) reported consonance between their role in the facility and their job description. However, 46% (95% CI: 33.7%–58.1%) of staff members reported that they perform tasks outside their scope of practice. Of all the tasks said to be outside their scope of practice, wound care was the task most reported (by 17 ENAs and caregivers), followed by the administration of medication and the insertion of a NGT, which were both reported by four ENAs and caregivers.

### Satisfaction

Of the 57 patients and families interviewed at follow-up, 25 respondents (44%; 95% CI 31-58%) indicated they were satisfied with the care received and did not suggest areas of care that needed to be improved in IC. Seven participants wanted more senior nurses and PTs. One participant also felt that transport needed to be improved between IC and their referring hospitals when they go for their outpatient clinic appointments and felt that government needed to cover the cost of this transportation instead of it being for the patient’s account. Furthermore, from the admission pattern, the IC service is providing IC since LoS is consistent with how one understands IC.^6-16^ Only 3 respondents (5%; 95% CI: 1%-15%) wanted an increased IC LoS.

When patients or families were interviewed post-discharge and were asked to compare the quality of care they received, 66% reported that care at the IC facility was better, 23% reported it was the same quality and 11% reported it was worse than care at the referring institution. The three most common reasons given why most felt that care was better at the IC facility (n = 41) was the caring and friendly staff (n = 20 or 49%), the presence of physiotherapy (n = 7 or 17%) and the practice of wound care (n = 2 or 5%).

### Patient Outcomes

Fifty-seven patients were traced on follow-up and the median time to survival was 107 days. Of these, 21(37%) had died. There was no statistical difference in the risk of death between those admitted in the palliative care section and the SACW (*P* = .57. One third of the deaths (n = 7) occurred during their admission at IC and the rest occurred after discharge from IC. While 50% of those admitted in the SACW were expected to survive beyond 107 days the largest survival estimate that could be determined with accuracy among palliative care ward patients is the 25th percentile (69 days).

Table 6 shows that in the model which included only the presence of a Care-plan (Referring Institution Care-plan and IC Discharge Care-plan) as a predictor of survival, those with an IC Discharge Care-plan had a 62% statistically significant lower risk of death than those with only a Referring Institution Care-plan (hazard ratio 0.38; CI: 0.15-0.97). When the other variables were added to the model, the risk of death was still reduced (58%) but the Hazard Ratio was no longer statistically significant (hazard ratio 0.41; 95% CI: 0.15-1.16). A stroke diagnosis was found to confound the association between the presence of an IC Discharge Care-plan and survival; age was not, however, found to be a confounder. Although the presence of a stroke (versus non-stroke) and a CHW visit (versus no CHW visit) were associated with lower risks of death, these hazard ratios (0.53 [95% CI: 0.19–1.51] and 0.77 [95% CI: 0.23–2.53], respectively) were not statistically significant.

**Table 6:** Cox’s Proportional Hazard Model on Predictors of Survival

**Predictors**	**Model With All Predictors**	**Model With Only Care-Plan**
	**Hazard Ratio (95% CI)**	**Hazard Ratio (95% CI)**
IC discharge Care-plan	0.42 (0.15–1.16)	0.38 (0.15–0.97)
Age^a^ (10 years)	1.12 (0.83–1.51)	
Stroke	0.53 (0.19–1.51)	
Confirmed CHW visit	0.77 (0.23–2.53)	
	*P* = .25	*P* = .06

Abbreviations: IC, intermediate care; CHW, community health worker. ^a^ Represents a 10-year increase in age.

Of the 47 patients (82.5%) who reported to have accessed healthcare post-discharge from IC, 10 received PT and OT and 2 received speech therapy. Even though three patients who needed health services post-discharge from IC made use of traditional healing many patients continued to receive ‘W*estern medical care*’ in the form of a CT scan (n = 2), emergency operation (n = 2), re-admission to an acute hospital (n = 2), re-admission to IC (n = 1) and making use of psychological services (n = 1). Only 15 of the 59 (33%) patients who could have been visited by a CHW had a confirmed CHW visit after their discharge from IC.

## Discussion

### The Model of Care in the Health System

**Figure 2:**
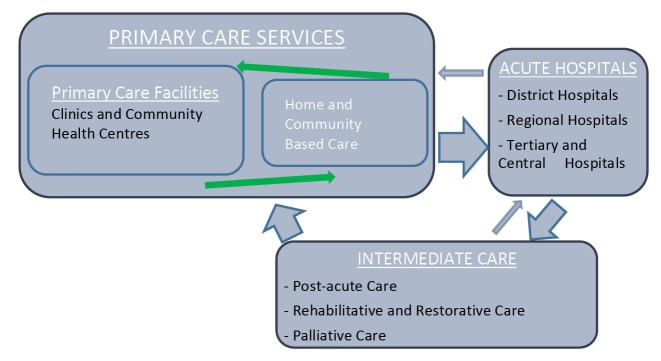
An Ideal Continuum of Care Pathway in a Health System.

This study was conducted at a time when the WC Provincial DoH was reviewing its policy^21^ and developing a more accurate description of the mixture of services offered in non-acute services (de-hospitalised care) framed as IC. We found that IC is a vital but often sidelined component of the health system that offers care in 6 main domains as shown in Table 1. Kane^8^ suggests that IC should be viewed as an extension of acute hospital care whose care is mostly rehabilitative. This situation may, however, not always be practical in LMIC due to resource constraints and the disease burden.^18^ This study is a classic example, where IC services comprised of respite, rehabilitative and minimal palliative care services. Internationally, there seems to be agreement that IC expedites discharge from hospital.^6-16^ However, unlike in the United Kingdom where IC may prevent admission to hospital,^14^ in a LMIC context patients have to be referred from a hospital (Figure 2).

With a bed capacity of 106-beds, the fact that 105 of these beds were occupied in a 30-day period is indicative of the demand for IC beds. This is further supported by the bed utilisation rate of 94% reported for the hospital for the period 01 April 2011 to the 31st of March 2012. The high reported patient satisfaction further suggests that patients and their families appreciate the care they received while at IC, mainly attributable to caring staff. A complex set of skills is key to ensuring that the wide range of care-needs required by patients who access IC are met.

In the WC, IC is poised to form part of integrated care pathways that link community-based primary care and acute hospitals.^8,21,22^ Figure 2 depicts the relationship between different levels of service and IC wherein patients are referred into IC from acute hospitals and could be referred back to acute hospitals if they worsen, develop complications or require routine follow-up.

Under normal circumstances however, IC acts as an intermediary that maintains the continuity of care by ensuring a successful discharge of patients into the community or other appropriate environment such as an old age home or other institutions.^21,22^ On discharge from IC patients should have a clear discharge plan which details a referral to HBC and other facets of primary care.^21,22^ Outpatient appointments at referring institutions would have been issued at the institution which initially referred the patient to IC. Given this, it is therefore imperative to map the service against this idea, starting with a full understanding of the patients who utilise this service.

### Who Are the Patients?

There were some important demographic and disease patterns that differed from that found in health systems in the north. While the gender parity found in admissions to IC is consistent with international literature,^11,30,31^ the average age in our sample (56 years) was generally lower than that of IC users in developed nations.^11,12,15,32^ This likely reflects the different disease burden in South Africa^32^ (particularly a high HIV/TB burden concentrated in younger adults) and more poorly controlled hypertension resulting in earlier onset of stroke.^32^ In this study, most patients in the palliative care section (79% or n = 11) had HIV/AIDS and TB, and were younger (41 years compared to 60 in the SACW).Gender could not explain the age differences as there was no difference in age by gender.

The marital status data of patients was consistent with the hypothesis by Kane and colleagues^33^ which expects IC users to mostly be unmarried because spouses serve as “informal caregivers.”^11,34^ However, despite most participants being unmarried, there was a median of 2 adults per household and most had someone to care for them when ill; this could be explained by the fact that poorer households are likely to be over-crowded.^35^ Nonetheless, more than a third of participants stayed alone and the family member who cares for them was reported to live in a different household from them, which supported the need for increasing the availability of CHW services post-discharge from IC.^36^

Although homelessness affected a minority of patients, it is of concern. This not only subjects individuals to unhealthy living conditions but also renders continuity of care difficult. Investigators failed to make contact with all 4 homeless participants, as compared to the 89% (n = 57) of non-homeless participants traced on follow-up. Access to healthcare for such individuals can only be guaranteed if the health services have regular and structured working relationships with other government departments responsible for welfare and social development.

Stroke was the most prevalent primary condition among admitted patients, a phenomenon consistent with international literature.^9,11,16,31-33^ This high stroke burden could signify poor care in the community. Furthermore, hypertension was prevalent in 75% of all stroke patients and almost 85% of the patients sampled had at least one chronic condition. The literature generally attributes hypertension to be a risk factor in over 60% of stroke patients and as much as 86% among IC patients.^37,38^

### Access to Intermediate Care

Close residential proximity to IC facilities and referral from a teaching hospital are 2 associations with admission described in literature^11,39^ that were evident in the study findings.Most referrals (62%, n = 42) into IC in this study were from 2 nearby secondary and tertiary teaching hospitals situated in the same sub-district as the IC facility meaning there was relatively poor access for patients from other districts and sub-districts. Moreover, although most patients in this study were of lower socio-economic status, they came from less disadvantaged sub-districts of the City of Cape Town Metro, suggesting a problem of inequity of access to IC at district level.

The narrow definition of IC that existed at the time of the study (defining IC as mainly respite, restorative and rehabilitative care)^7^ could have influenced the way referrals were implemented and the seeming inequity in access. For example, one of the sub-districts had a palliative care centre, which did not treat subacute patients who were expected to be referred to the study IC facility. This may have created confusion since referrals do not seem to have been well implemented in our study. The difference in referral might have also arisen as a result of increased awareness of the value of IC in teaching hospitals.^11^ The new policy will widen the definition of IC and so help to relieve inequity in care. For example, prior to the policy review, palliative care was available at only a handful of facilities but following the policy review, patients requiring palliative care can now access services at any of the 25 IC facilities in the province.

Burdens posed by failure elsewhere in the health system could result in a situation where patients experience barriers to access healthcare services at the facilities nearest to them.^33^ This was evidenced in this study with a few patients who travelled from a different province, the Eastern Cape, to the WC for healthcare. The challenge is that migration of patients will adversely impact the continuity of care plan especially if these patients plan to return to their Provinces of residence after discharge from IC.

These geographic and economic access factors strengthen the views that those admitted to teaching hospitals, those whose hospitals are closest to IC and those who reside in higher income communities will benefit more from IC services as they are more likely to be referred.^9,11,34^ It is therefore important for the health system to put structures in place for monitoring equity in access and to prioritise access for those currently excluded.

Inequity in access to IC may be aggravated by financial obstacles.^9^ Even when already admitted to IC, transport for follow-up back to the referring hospital was largely at the patient’s own cost. Studies in South Africa^40,41^ have suggested that transport costs contribute the largest proportion of direct patient costs. Employment levels amongst respondents were low which meant that out-of-pocket payments are likely to be a significant obstacle to patients accessing referring hospital services whilst admitted. IC facilities should therefore provide free transport for patients during admission to prevent this barrier to access, a recommendation consistent with that of the IC policy task team in the WC.^21,22^

Poorly developed referral systems and poor discharge planning are structural barriers to access to health services.^8^ Even though a large proportion of the IC inpatients had been admitted in a health facility on the year preceding the study, only 13 patients (21%) had used a CHW ever before and only 25% (n = 15) of the discharged patients had a confirmed CHW visit post-discharge. This does not advance the vision of Alma-Ata to rely “…at local and referral levels, on health workers, including…community workers….”^1,2^ This unrealised vision of service integration, present in many countries including South Africa,^1,2^ can only be achieved with a coherent continuity of care pathway from the IC to the community and adequate utilisation of CHWs.^8^

Poor use of CHWs is common in literature,^36^ and may arise because referring hospitals discharge patients without a clear Care-plan; alternatively, IC staff might assume that referral to HBC is the responsibility of the primary referring institution.^11^ Staff at IC facilities therefore need to recognise that discharge planning should include HBC referral as part of quality care.^42^

### Patient Care Needs

As evidenced in Table 5, patients mostly received nursing (urinalysis, oxygen administration, etc), specialised care (eg, wound care, catheter insertion, etc) and rehabilitative care. The social work and ADLs are likely to have been under-reported because they were likely considered to be routine.^43^ For instance, dressing or feeding a patient (ADL) was not always recorded.

The results suggested that staff largely saw the needs of their patients as consistent with the skills they have, but there was some degree of inconsistency of staff views comparing what skills they have to what they actually do. Moreover, according to staff, patients need more ADLs, rehabilitation and social work care than staff can provide (Table 5), which suggests a need to strengthen these skills through training and up-skilling in these areas of care. This is also in the context of a large proportion of patients (63% or n = 43) who received rehabilitation care during their admission at IC.

Although wound care is considered to require specialised nursing care, it featured as the most prominent task that caregivers and ENAs reported performing outside their scope of practice and it has an unavoidable presence in IC.^42^ For example, about a quarter of patients required post-surgical wound care and 12% of patients had pressure ulcers. The literature acknowledges^44^ that even though pressure ulcers should be preventable, it is impossible in practice to prevent all pressure ulcers.^44^ The prevalence of 12% in this study is consistent with rates found in the United States (12%), Germany (7%), and Canada (14%) in IC.^44,45^

Caregivers have a key role reducing pressure ulcer prevalence to the lowest rate possible, through identification of risk factors for pressure ulcers including older age, cognitive impairment, physical impairments and impaired sensory sensations^44,45^ and awareness of the latest preventive techniques.^44,45^ It is, however, also encouraging that, in this study, staff felt that a skilled caregiver can manage wound care without supervision, a view supported by literature.^42^

A large proportion of patient respondents (n = 20 or 49%) and their families favoured IC over their referring institution mainly because of the caring staff, the rehabilitation received in-care (n = 7 or 17%) and the wound care (n = 2 or 5%). Twenty-six percent (n = 12) of patients who sought healthcare after discharge from IC reported continuing with rehabilitative care, which further emphasises the importance of rehabilitative care after an acute illness.

### Human Resources

Sceptics who argue against the inclusion of IC facilities in healthcare have often based their criticism on the lack of patient monitoring to detect complications in IC facilities.^46^ The presence of a sessional or on-call medical professional and PNs at the study site partly addressed this concern as these professionals will be able to screen, assess, prescribe, administer medication and refer to the appropriate level of care when complications are suspected. They also have the ability to suggest a change of medications if drug-related complications are suspected or if there is no improvement on the initially prescribed treatment.

A new cadre of mid-level worker can be trained on the basic care of diabetic and hypertensive patients by empowering them with skills to monitor glucose, administer insulin (subcutaneous), conduct urinalysis and monitor blood pressure regularly. Because of the high risk of death amongst IC patients, competency in bereavement counselling would also be required for staff.

However, mid-level workers cannot substitute for professional therapists (such as OTs, PTs and speech therapists) as midlevel workers do not have the ability to identify patients at risk of developing complications such as an embolus or other complications of coronary artery disease.^46^ Nonetheless, mid-level workers with combined rehabilitation skills (physiotherapy, occupational therapy and speech therapy) can be trained to safely care for patients under professional guidance. Following the IC policy review^21,22^ the WC Provincial DoH introduced a new cadre of mid-level workers known as RCWs to help increase rehabilitation capacity in IC.

However, the experience of training clinical associates, a cadre of health worker equivalent to a physician associate in the United States, which commenced in 2008, is noteworthy.^47^ There are still no career pathways and there are fears that clinical associates may not be able to work without adequate supervision.^47^ For that reason, the introduction of a new IC mid-level cadre should ensure that the career pathways and scope of practice are simultaneously developed.^47^

### Patient Outcomes

A key finding of this study consistent with literature^46,48^ was that IC patients have a high mortality (37% at follow-up amongst those traced). Also consistent with literature^43^ and the study hypothesis is the suggestion that a confirmed CHW visit (though not statistically significant) and the presence of a Care-plan were associated with a lower risk of death. Whilst these findings were not statistically significant in the multivariate model with all four predictors included, the presence of a Care-plan alone was found to be significantly associated with a 62% reduction in the risk of death (hazard ratio: 0.38; 95% CI: 0.15-0.97).

The lack of statistical significance for the protective effect of a confirmed CHW visit may be due to limited study power because of a small sample size. Also having a Care-plan could be an indicator of staff views on whether a patient actually needed one (ie, their prediction that a patient would survive), rather than itself being a predictor of survival and so may be a reflection of an association rather than a causal relationship. Nonetheless, it is plausible to anticipate that a Care-plan that extends beyond an IC facility will be likely to have a beneficial impact on mortality.^43^

### How Subacute Care Was Understood by Clients and Staff

Optimal utilisation and benefit from IC services is dependent on the understanding amongst those staff who refer to and work in IC, and amongst patients and family. Even though this study did not assess the appropriateness of the referral to IC, literature describes a phenomenon where patients are either referred to IC prematurely or are referred without a Care-plan.^30,42^ Other studies will however, need to be conducted to assess the appropriateness of referrals to the IC facility included in this study.

Patients and family understood this service as a caring environment that is primarily responsible for rehabilitation services. Since this is an understanding that is consistent with literature,^8,9,11,13,30,42^ it is important to inform referring institutions of this finding so that they can empower patients and their family by educating them that rehabilitative care is only one component of IC. The suggestion by IC staff that IC patients are likely to require ADL care followed by specialised nursing care and rehabilitative care could be interpreted as suggesting that the service mostly caters for convalescence, respite and rehabilitative care respectively. There was, however, no literature identified that could be used to compare the understanding of IC by staff.

## Limitations

The study has a number of limitations. Firstly, the findings cannot be extrapolated to other IC facilities elsewhere in the province or country but does help to highlight challenges experienced in this sector of care and how this sector of care relates with acute hospitals and HBC. Furthermore, the study only included patients for a one month period which limits generalisability to the population of patients who access the IC facility at other times. Nonetheless, there is no reason to anticipate admissions in this period would have systematically differed from admissions during other periods of the year.

Secondly, the response rate of 65% among patients was less than desirable and could have resulted in a response bias if participants not interviewed were statistically different from the participants. For example, patients who were more ill might differ systematically from other patients. Case mix and multimorbidity have a strong impact on patient outcome eg, survival, this could not however be tested because of the low power of the study. However, the reasons for non-response were largely beyond the researchers’ control and were logistic, rather than selection or refusals. Moreover, the study had a good retention rate amongst participants (84%; 95% CI: 73%-92%).

Thirdly, a large section of the in-patient questionnaire was adopted from the ICF^28^ which is standardised and validated for a South African population.^28^ An information bias could have, however, resulted as a result of the translation into local languages. It is also recognised that, even though unlikely, the assumption that clinical notes were more accurate than clinical information reported by patients could have resulted in an information bias and thus misclassified patient care needs and diagnoses.

Fourthly, even though the categorisation of staff skills and patient needs was largely based on literature, some of the functions could not be located in literature and this could have thus affected the reliability of this categorisation. Furthermore, the study subjectively uses service provision as a proxy for patient needs and does not address unmet needs such as poor pain control, which might have affected the validity of the findings. However, the high patient satisfaction reported with the IC service compared to the referring hospital service suggests it is unlikely that lack of pain control was a major unrecorded factor in this study.

Lastly, it was also initially anticipated that the follow up appointments would be in the form of a home visit but this was not possible due to logistical reasons. Even though the telephonic interview could have indirectly prevented a social desirability bias, some participants might have had difficulty conveying personal information on the phone. This is however, balanced by the fact that all participants were followed up in the same way.

## Conclusion

The increasing life expectancy and quadruple burden of disease facing South Africa^32^ requires a coherent and well-structured health system with clear pathways accessible to all in need. Such a system requires a comprehensive PHC approach with clearly defined roles and referral pathways for CHWs/HBC, primary care facilities, acute hospitals and IC facilities. In this way the vision of access to healthcare of the Alma-Ata conference in 1978 will also be realisable as an achievable vision in the 21st century.

Finding from the study support the hypothesis that developing a Care-plan which extends beyond a hospital admission could improve the quality of life and reduce mortality after discharge from IC. The data also suggest potential for mortality reductions from including CHWs in this Care-plan. Future studies should investigate the reasons for the skewed referral pathway identified in this study, where the majority of patients at IC are referred from secondary and tertiary hospitals within the same sub-district and should examine in more detail the question of equity in access to IC.

Study findings also suggest that mid-level workers could be usefully incorporated into IC care. They should be equipped with skills in ADL, social support, bereavement counselling, communication, and basic speech therapy, OT, physiotherapy, wound care and nursing skills. A major human resource management challenge is to have a clearly defined scope of practice for all staff members as there currently are roles potentially outside their scope of practice.

Most important though, would be the recognition by health workers, policy-makers and other stakeholders that IC services are not an optional form of care but an integral service within the health system.

This study contributed to the provincial IC policy document by providing insight into the patient and staff profile of the study site and the extent and nature of articulation of services with IC care. Now that the new IC policy has been adopted it is therefore hoped that packages of care delivered through IC can be better monitored in future.

## Acknowledgements

We would like to thank patients, families, staff and management of Booth Memorial Hospital for granting us permission to conduct this study at the facility. My gratitude will not be enough if I forget to thank the assistance provided by Ms. Tobeka Qukula, Dr. Tracey Naledi and the Western Cape Provincial Department of Health’s Health Impact Assessment Unit, Professor Jennifer Jelsma, Dr. Joey Cupido, Mr. Rauf Sayed, Dr. Vivien Essel, Ms. Gugu Msithini, and Dr. Liz Gwyther; your contribution to my success has not gone unnoticed.

## Ethical issues

Ethics approval was granted by the UCT Faculty of Health Sciences, with Ethics approval number HREC REF: 265/2011. Informed consent was obtained from all study participants prior to their participation. Family members legally entitled to do so, gave consent and participated on behalf of patients who could not communicate.

## Competing interests

The authors declare that they have no competing interests.

## Authors’ contributions

The Principal Investigator (SAM) planned, designed, executed, analysed and did the main drafting of the manuscript and its revisions and signed off on the final version. LL contributed to the design of the study, assisted with editing the manuscript, commented on revisions and signed off on the final version. DP contributed to the design of the study, assisted with editing the manuscript, commented on revisions and signed off on the final version.

## Authors’ affiliations

^1^Public Health Department, Walter Sisulu University, Mthatha, South Africa. ^2^School of Public Health and Family Medicine, University of Cape Town, Cape Town, South Africa. ^3^Western Cape Department of Health, Cape Town, South Africa.

## Key messages

**Implications for policy makers**

From our research, policy-makers will be able to better:

- The findings of this research help to clarify the definition of intermediate care (IC).
- A policy on IC is needed in the health system along with the development of appropriate skills and packages of care for IC facilities.
- There is evidence of differences in referral patterns to IC, and referral from IC to community-based services, which reflect inequities in care. This requires efforts to ensure that all health workers are aware of and make use of the services offered by IC facilities and for community-based follow up.
- Health workers, policy-makers and other stakeholders need to recognise that IC services are not an optional form of care but an integral service within the health system.

**Implications for public**

It is not always possible for hospitals to discharge a patient directly to their home. Patients may only be partially recovered or their family is not in a position to care for them while they recover. After a stroke, for example, the patient might need to adapt to their environment differently on recovery and they need health workers to help them with this recovery, such as rehabilitation therapists, nurses and some care from medical doctors and pharmacists. intermediate care (IC) facilities are therefore different from most hospitals and provide care more advance than home-based care (HBC). These types of facilities should be made available and awareness of their services increased. This study described one example of an IC service in Cape Town, South Africa. The study suggested that better matching of provider skills to the needs of patients, formulating a Care-plan on discharge and better integration between referral and community-based services can improve the quality of IC and contribute to positive patient outcomess.
